# Predictors of high-cost patients with acute whiplash-associated disorder in Japan

**DOI:** 10.1371/journal.pone.0287676

**Published:** 2023-06-28

**Authors:** Kazuhiro Hayashi, Kenji Miki, Tatsunori Ikemoto, Takahiro Ushida, Yukiko Shiro, Tomoko Tetsunaga, Toshifumi Takasusuki, Masako Hosoi, Masao Yukioka

**Affiliations:** 1 Department of Physical Therapy and Rehabilitation Science, University of Iowa, Iowa City, IA, United States of America; 2 Multidisciplinary Pain Center, Aichi Medical University, Nagakute, Japan; 3 Center for Pain Management, Hayaishi Hospital, Osaka, Japan; 4 Faculty of Health Science, Osaka Yukioka College of Health Science, Ibaraki, Japan; 5 Department Rheumatology, Yukioka Hospital, Osaka, Japan; 6 Japan Pain Foundation, Tokyo, Japan; 7 Department of Orthopedic Surgery, Aichi Medical University, Nagakute, Japan; 8 Institute of Physical Fitness, Sports Medicine and Rehabilitation, Aichi Medical University, Nagakute, Japan; 9 Department of Pain Medicine, Aichi Medical University, Nagakute, Japan; 10 Department of Physical Therapy, Faculty of Rehabilitation Sciences, Nagoya Gakuin University, Nagoya, Japan; 11 Department of Orthopaedic Surgery, Okayama University, Okayama, Japan; 12 Department of Anesthesiology, Dokkyo Medical University, Mibu, Japan; 13 Department of Psychosomatic Medicine and Multidisciplinary Pain Center, Kyushu University Hospital, Fukuoka, Japan; Al Mansour University College-Baghdad-Iraq, IRAQ

## Abstract

**Introduction:**

The proportion of neck injuries due to traffic accidents is increasing. Little is known about high-cost patients with acute whiplash-associated disorder (WAD). The present study aimed to investigate whether time to first visit for conventional medicine, multiple doctor visits, or alternative medicine could predict high-cost patients with acute WAD in Japan.

**Methods:**

Data from a compulsory, no-fault, government automobile liability insurance agency in Japan between 2014 and 2019 were used. The primary economic outcome was the total cost of healthcare per person. Treatment-related variables were assessed based on the time to first visit for conventional and alternative medicine, multiple doctor visits, and visits for alternative medicine. Patients were categorized according to total healthcare cost (low, medium, and high cost). The variables were subjected to univariate and multivariate analysis to compare high-cost and low-cost patients.

**Results:**

A total of 104,911 participants with a median age of 42 years were analyzed. The median total healthcare cost per person was 67,366 yen. The cost for consecutive medicine, for consecutive and alternative medicine, and total healthcare costs were significantly associated with all clinical outcomes. Female sex, being a homemaker, a history of WAD claim, residential area, patient responsibility in a traffic accident, multiple doctor visits, and visits for alternative medicine were identified as independent predictive factors for a high cost in multivariate analysis. Multiple doctor visits and visits for alternative medicine showed large differences between groups (odds ratios 2673 and 694, respectively). Patients with multiple doctor visits and visits for alternative medicine showed a significantly high total healthcare cost per person (292,346 yen) compared to those without (53,587 yen).

**Conclusions:**

A high total healthcare cost is strongly associated with multiple doctor visits and visits for alternative medicine in patients with acute WAD in Japan.

## Introduction

The incidence of head or neck injury is high in traffic accidents [1–3]. A traffic accident involving rapid acceleration or deceleration triggers whiplash-associated disorder (WAD) with bony or soft-tissue injuries [4]. WAD, triggered by an accident with an acceleration or deceleration mechanism, is defined based on the severity of signs and symptoms on a 4-point scale [4]. WAD can present a variety of symptoms, including neck pain or stiffness, headache, radicular symptoms, and cognitive impairment [4]. Pain and disability symptoms rapidly decrease in the initial months after the accident but show little improvement after 3 months [5]. About 50% of patients with acute WAD recover within a year, while in the rest there is remaining pain or disability [6]. A delayed recovery from WAD is associated with older age, injured regions, and the type and intensity of clinical care [7–10].

Economic evaluation is a potentially useful decision-making tool for clinical practice, management, or health policy [11]. The annual economic cost of pain varies between $560–635 billion, greater than the annual costs of heart disease ($309 billion), cancer ($243 billion), and diabetes ($188 billion) in the United States [12]. Half of the pain-associated cost is for direct healthcare ($261–300 billion) [12]. Among them, the annual cost of WAD is estimated to be approximately $2.7 billion in the U.S. [13].

The characteristics of high-cost patients have been investigated for over half a century in various diseases [14, 15]. Diseases of the musculoskeletal system and connective tissue are often associated with high cost [15, 16]. Further, population studies on the factors associated with the healthcare cost of pain, including 12985 patients with musculoskeletal pain, 7792 individuals reporting pain, 5834 older women with arthritis, and 37800 patients with low back pain [17–20], suggested that high cost is associated with older age, male sex, comorbidities, worse symptoms, non-employment, supplementary health insurance, residence in an urban area, and alternative medicine use [17–20]. Two population studies [21, 22] in Australia found that high cost in WAD was associated with middle and older age, female sex, accidents in the latest year of the study period, and lodgment of common-law claim. However, little is known about treatment-related variables of high-cost patients with acute WAD.

The number of doctors’ consultations in Japan are the first or second highest in the world, 50% more than in Australia [23]. Overtreatment in the initial phase after acute WAD is associated with slow recovery [7, 8, 10, 24]. Moreover, there are numerous alternative medicines available and covered by national health insurance in Japan [25]. Alternative medicine is sought as much or more than a third of conventional medicine in Japan for musculoskeletal disorders. The major types of alternative medicine for musculoskeletal disorders in Japan are judo therapy (a type of manual therapy), acupuncture, moxibustion, and massage therapy [26, 27]. Japan’s automobile insurance under a government compulsory insurance system, the General Insurance Rating Organization of Japan, covers conventional medicine and a judo or massage therapist as alternative medicine [28].

The present study aimed to investigate whether time to first visit for conventional medicine, multiple doctor visits, or alternative medicine could be used to predict high-cost patients with WAD using no-fault government insurance claims data in Japan.

## Methods

### Data source

Anonymous data from a compulsory, no-fault, government automobile liability insurance agency, the General Insurance Rating Organization of Japan, were used. The General Insurance Rating Organization of Japan undertakes the necessary procedures for residual disability claims under the government compulsory insurance system, providing continuous insurance for 92.3% of all automobiles in Japan, regardless of the accident risk level of each driver [28]. The General Insurance Rating Organization of Japan covers conventional and alternative medicine and disability claims for patients of automobile accidents. Conventional medicine was defined as usual medicine practiced by medical doctors. Alternative medicine was defined as treatment by a judo or massage therapist in the insurance.

### Participants

The inclusion criteria were as follows: (1) involvement in a car crash with rear-end collision, contact with a vehicle moving in the same or opposite direction; (2) primary diagnosis of acute (<3 months) WAD Grade 1 or 2 following the crash [4]; (3) treatment in a medical institution covered by compulsory automobile liability insurance; (4) end of the treatment between June 2014 and December 2019; and (5) <400,000 yen in the amount of loss without residual disability (a category of small amount in no-fault government insurance in Japan) or <1,200,000 yen in the amount of loss with residual disability (minor residual disability according to no-fault government insurance in Japan). The residual disabilities ranged from levels 1–14, with 14 being the most minor.

The exclusion criteria were as follows: (1) victims of car crash wherein the claim had not been closed, (2) missing data of treatment or traffic accident-related variables, and (3) patients with fractures, dislocations, or spinal cord injuries. This study was approved by the Ethics Committee of the Osaka University Graduate School of Medicine (No. 17136). Requirement for written informed consent was waived by the Ethics Committee owing to the design of the study.

### Data collection

The primary economic outcome was the total healthcare cost per person, which included the cost of consecutive and alternative medicine, payment for pain and suffering, and miscellaneous expenses. The cost of consecutive and alternative medicine was calculated in the claim for compulsory automobile liability insurance in Japan [28]. The payment for pain and suffering was calculated based on treatment and residual disability in Japan [28]. The cost is shown in Japanese yen per patient (approximately 130 yen per USD $1). Patients were categorized into the following three groups according to percentiles of total healthcare cost: low-cost (lower third: <42,448 yen), medium-cost (medium third: ≥42,448 yen, <121,464 yen), or high-cost (upper third: ≥121,464 yen).

The predictive variables were patient characteristics, and traffic accident- and treatment-related variables. Patient characteristics were collected based on patient age, sex, occupation, history of WAD claims, and resident area. Occupation was classified into salaried workers, homemakers, students, and others, including self-employed and unemployed workers. The resident areas of patients were classified into urban (>1000 people per km^2^), suburban (200–1000 people per km^2^), and rural (<200 people per km^2^) in each prefecture. The total healthcare cost per person was compared according to prefectures’ characteristics, economic status, medical status, and traffic status in the National System of Social and Demographic Statistics of Japan [29]. Traffic accident-related variables were assessed based on collision type, engine size of the other vehicle, and responsibility of the patient. The total weight of the other vehicle was recorded as a part of the traffic accident report. Collision types were classified as follows: rear-end collision, contact with a vehicle moving in the same direction including contact side to side, or contact with one moving in the opposite direction. Treatment-related variables were assessed based on the time to first visit for conventional and alternative medicine, multiple doctor visits (different medical institutions), and visits for alternative medicine. Conventional medicine was defined as treatment by a physician. Alternative medicine was defined as treatment by a judo or massage therapist.

Clinical outcomes were the number of visits for conventional and alternative medical institutions, duration of conventional and alternative medicine, time to compensation closure, and chronicity more than 3 months after the traffic accident. The duration of medical interventions has been verified as a valid marker of health recovery [7–9, 30]. As compensation is calculated as the number of treatment visits in Japan, this value was recorded as well [31].

### Statistical analysis

The normality of the distribution for each measure was evaluated using the Shapiro–Wilk test for continuous variables. Continuous variables were presented as medians and interquartile ranges (IQRs), while categorical variables were presented as the number and percentage of patients.

Patient characteristics and traffic accident- and treatment-related variables were included in the multivariate logistic regression analysis for the low- and high-cost groups. Odds ratio (ORs) and 95% confidence interval (CIs) were calculated for all variables via multivariable logistic regression analysis to estimate predictive factors for total healthcare cost. The association between variables was analyzed using Spearman’s rank correlation coefficient (CC). Patients were also compared based on multiple doctor visits and visits for alternative medicine, using the Kruskal–Wallis test or chi-squared test.

All data were analyzed using SPSS Statistics (version 26.0; IBM, Armonk, NY). All statistical tests were two-tailed and p < 0.05 was considered statistically significant.

## Results

**Fig 1:**
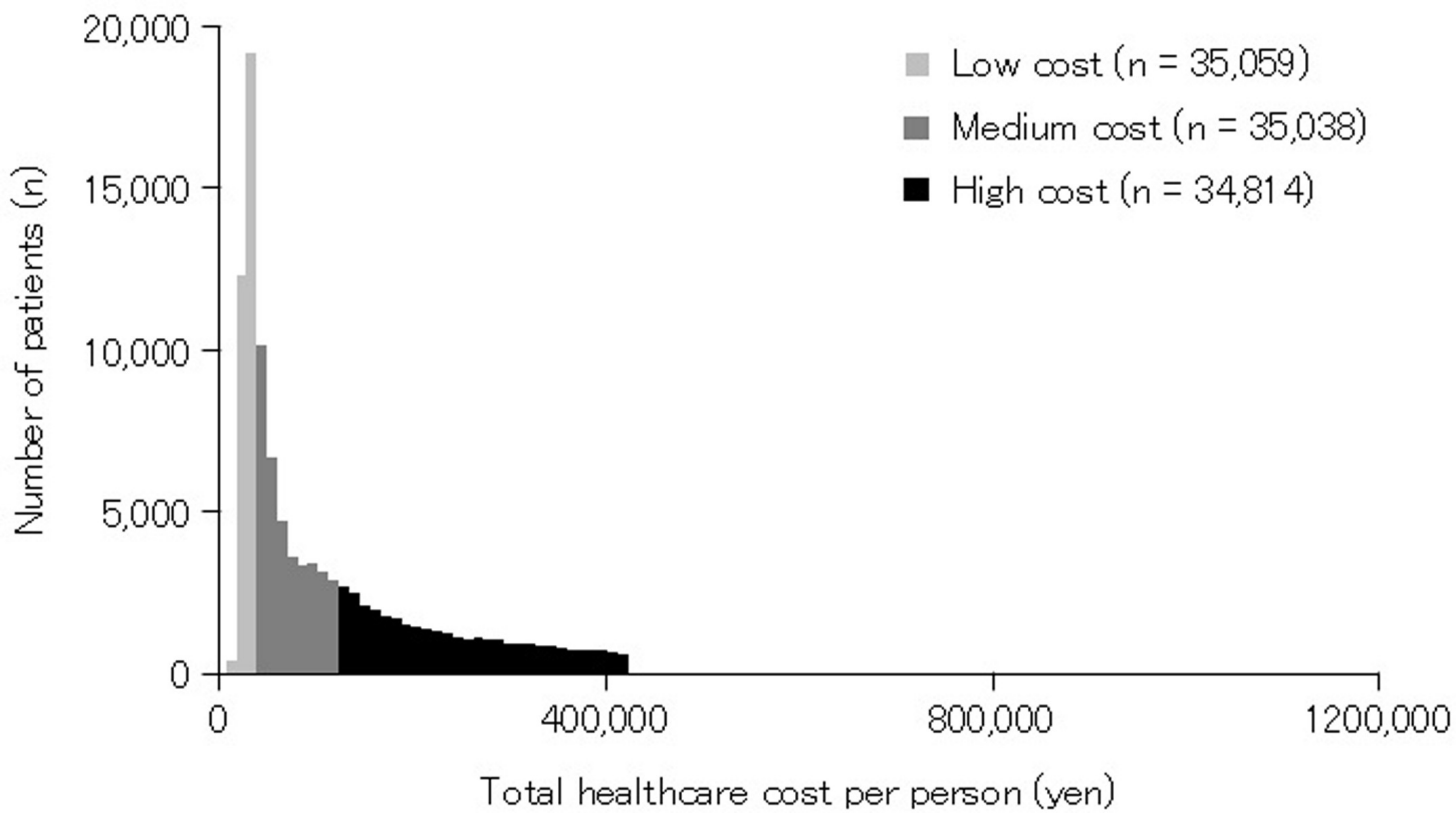
Distribution of total healthcare cost per person. The median total healthcare cost per person was 67,366 yen (IQR: 36,420–158,786 yen). Patients were categorized into the following three groups according to percentiles of total healthcare cost: low-cost (lower third: <42,448 yen), medium-cost (medium third: ≥42,448 yen, <121,464 yen), or high-cost (upper third: ≥121,464 yen).

Data from a total of 104,911 participants (56,931 males and 47,980 females) with a median age of 42 years (IQR: 27–56 years) were included in the analysis. Patient characteristics are presented in Table 1. The median total healthcare cost per person was 67,366 yen (IQR: 36,420–158,786 yen; Fig 1). The median time to compensation closure was 19 days (IQR, 9–46 days). A total of 8901 (8%) and 7735 (7%) patients visited multiple doctors and alternative medicine, respectively. The total healthcare cost per person in each resident prefecture was significantly associated with the unemployment rate (CC = 0.300; p = 0.040), number of traffic accidents (CC = 0.473; p = 0.001), and persons killed or injured by traffic accidents in the prefecture (CC = 0.481, p = 0.001) (S1 Table and S1 Fig). Population, natural environment, education, safety, economic status, and medical status were not associated with the total healthcare cost.

**Table 1:** Participants’ characteristics.

	Overall	Low cost	Medium cost	High cost
	(n = 104,911)	(n = 35,059)	(n = 35,038)	(n = 34,814)
*Demographics*				
Age (years)	42 [27–56]	39 [23–55]	42 [27–56]	44 [31–57]
Age (group), n (%)				
17 ≤	11,388 (11%)	6,346 (18%)	3,402 (10%)	1,640 (5%)
18–39	35,898 (34%)	11,280 (32%)	12,462 (36%)	12,156 (35%)
40–59	36,157 (34%)	10,520 (30%)	11,989 (34%)	13,648 (39%)
≥ 60	21,468 (20%)	6,913 (20%)	7,185 (20%)	7,370 (21%)
Gender, n (%)				
Female	47,980 (46%)	15,587 (44%)	15,887 (45%)	16,506 (47%)
Male	56,931 (54%)	19,472 (56%)	19,151 (55%)	18,308 (53%)
Occupation, n (%)				
Salaried worker	57,609 (55%)	18,056 (52%)	19,624 (56%)	19,929 (57%)
Homemaker	15,648 (15%)	4,035 (12%)	5,300 (15%)	6,313 (18%)
Student	13,529 (13%)	7,130 (20%)	4,181 (12%)	2,218 (6%)
Others	18,125 (17%)	5,838 (17%)	5,933 (17%)	6,354 (18%)
A history of WAD in traffic accident, n (%)				
With	6,499 (6%)	1,429 (4%)	1,865 (5%)	3,205 (9%)
Without	98,412 (94%)	33,630 (96%)	33,173 (95%)	31,609 (91%)
Resident area, n (%)				
Urban	34,877 (33%)	10,308 (29%)	12,289 (35%)	12,280 (35%)
Suburban	42,598 (41%)	14,909 (43%)	13,728 (39%)	13,961 (40%)
Rural	27,436 (26%)	9,842 (28%)	9,021 (26%)	8,573 (25%)
*Traffic accident-related variables*				
Collision types, n (%)				
Rear-end collision	75,017 (72%)	25,896 (74%)	25,369 (72%)	23,752 (68%)
Contact with one moving in the opposite direction	13,364 (13%)	4,103 (12%)	4,309 (12%)	4,952 (14%)
Contact with a vehicle moving in the same direction	5,617 (5%)	1,637 (5%)	1,859 (5%)	2,121 (6%)
Others	10,913 (10%)	3,423 (10%)	3,501 (10%)	3,989 (11%)
Engine size of the other vehicle (cc)	1,348 [658–1,997]	1,339 [658–1,997]	1,386 [658–1,997]	1,348 [658–1,997]
A responsibility of the patient, n (%)				
With	13,076 (12%)	3,332 (10%)	4,291 (12%)	5,453 (16%)
Without	91,835 (88%)	31,727 (90%)	30,747 (88%)	29,361 (84%)
*Treatment related-variables*				
Time to first visit for conventional medicine (days)	1 [0–2]	1 [0–2]	1 [0–2]	1 [0–2]
Multiple doctor visits, n (%)				
With	8,901 (8%)	4 (0%)	2,381 (7%)	6,516 (19%)
Without	96,010 (92%)	35,055 (100%)	32,657 (93%)	28,298 (81%)
Alternative medicine, n (%)				
With	7,735 (7%)	18 (0%)	1,346 (4%)	6,371 (18%)
Without	97,176 (93%)	35,041 (100%)	33,692 (96%)	28,443 (82%)
*Clinical outcome*				
Number of visits for conventional medicine (days)	2 [1–6]	1 [1–1]	2 [2–4]	9 [5–14]
Number of visits for conventional and alternative medicine (days)	2 [1–7]	1 [1–1]	2 [2–4]	11 [7–17]
Duration of conventional medicine (days)	8 [1–32]	1 [1–1]	10 [3–22]	44 [24-72]
Duration of conventional and alternative medicine (days)	13 [3–40]	2 [1–4]	13 [6–26]	52 [33-80]
Time to compensation closure (days)	19 [9–46]	9 [8–11]	19 [11–31]	58 [39-86]
Chronicity, n (%)				
With	6,875 (7%)	63 (0%)	256 (1%)	6,556 (19%)
Without	98,036 (93%)	34,996 (100%)	34,782 (99%)	28,258 (81%)
*Economic outcome*				
Cost for conventional medicine (yen)	36,290 [23,900–69,355]	21,708 [17,919–25,240]	39,560 [31,611–51,2]	89,031 [64,460–121,855]
Cost for conventional and alternative medicine (yen)	40,192 [24,538–77,550]	21,710 [17,940–25,240]	40,638 [32,480–51,968]	98,619 [76,240–13,0473]
Total cost for health care (yen)	67,366 [36,420–158,786]	32,230 [28,128–36,450]	67,632 [52,250–92,830]	216,297 [159,333–298,117]
(Only patients with alternative medicine (n = 7,735))				
Time to first visit for alternative medicine (days)	6 [3–13]	2 [1–3]	6 [3–13]	7 [3–14]
Duration of alternative medicine (days)	34 [17–58]	1 [1–14]	10 [4–20]	40 [24–64]
Number of visits for alternative medicine (days)	10 [5–16]	1 [1–2]	4 [2–5]	12 [7–18]
Cost for alternative medicine (yen)	51,480 [30,590–79,520]	11,370 [8,172–14,920]	21,985 [15,465–28,630]	60,060 [40,910–86,230]

CI, Confidence intervals.

Data from continuous variables are shown in medians and interquartile ranges [IQR]. Data from categorical variables are shown in number and (%) of patients.

As shown in Tables 2 and 3, univariate and multivariate analyses based on total healthcare cost per person between the low-cost (median, 32,230 yen) and high-cost groups (median, 216,297 yen) revealed significant differences in demographics and traffic accident- and treatment-related variables. In multivariate analysis, female sex, being a homemaker, a history of WAD claim, residential area, patient responsibility in a traffic accident, multiple doctor visits, and visits for alternative medicine were identified as independent predictive factors for high cost (p<0.001). On the other hand, being a student and rear-end collision were identified as independent predictive factors for low cost (p<0.001). Collision types and engine size of the other vehicle exhibited no consistent association with the total healthcare cost. The ORs for high cost were high in multiple doctor visits (OR, 2673.269; 95% CI, 1002.247–7130.343; p<0.001) and visits for alternative medicine (OR, 694.289; 95% CI, 436.070–1105.414; p<0.001).

**Table 2:** Univariate logistic regression analysis for the high-cost groups.

	Odds ratio (95%CI)	p-value
*Demographics*		
Age (group)	1.014 (1.013–1.015)	p<0.001*
17 ≤	0.224 (0.211–0.237)	p<0.001*
18–39	1.131 (1.096–1.167)	p<0.001*
40–59	1.504 (1.458–1.552)	p<0.001*
≥ 60	1.093 (1.054–1.134)	p<0.001*
Female	1.126 (1.093–1.160)	p<0.001*
Occupation		
Salaried worker	1.261 (1.224–1.299)	p<0.001*
Homemaker	1.703 (1.632–1.777)	p<0.001*
Student	0.267 (0.253–0.280)	p<0.001*
Others	1.117 (1.075–1.162)	p<0.001*
A history of WAD in traffic accident	2.386 (2.238–2.544)	p<0.001*
Resident area		
Urban	1.309 (1.268–1.351)	p<0.001*
Suburban	0.905 (0.878–0.933)	p<0.001*
Rural	0.837 (0.809–0.866)	p<0.001*
*Traffic accident-related variables*		
Collision types		
Rear-end collision	0.760 (0.735–0.785)	p<0.001*
Contact with one moving in the opposite direction	1.251 (1.197–1.308)	p<0.001*
Contact with a vehicle moving in the same direction	1.325 (1.240–1.415)	p<0.001*
Others	1.196 (1.140–1.255)	p<0.001*
Engine size of the other vehicle	1.000 (1.000–1.000)	p = 0.580
A responsibility of the patient	1.768 (1.689–1.852)	p<0.001*
*Treatment related-variables*		
Time to first visit for conventional medicine	0.994 (0.991–0.997)	p<0.001*
Multiple doctor visits	2,017.973 (757.059–5,378.994)	p = 0.001*
Alternative medicine	436.050 (274.478–692.731)	p<0.001*
*Clinical outcome*		
Number of visits for conventional medicine	16.074 (15.018–17.205)	p<0.001*
Number of visits for conventional and alternative medicine	423.809 (272.919–658.123)	p<0.001*
Duration of conventional medicine	1.238 (1.234–1.243)	p<0.001*
Duration of conventional and alternative medicine	1.229 (1.225–1.233)	p<0.001*
Time to compensation closure	1.229 (1.225–1.233)	p<0.001*
Chronicity	128.877 (100.509–165.252)	p<0.001*
*Economic outcome*		
Cost for conventional medicine	1.000 (1.000–1.000)	p<0.001*
Cost for conventional and alternative medicine	1.001 (1.001–1.001)	p<0.001*
Total cost for health care	1.000 (0.998–1.003)	p = 0.766
(Only patients with alternative medicine (n = 7,735))		
Time to first visit for alternative medicine	1.141 (1.019–1.278)	p = 0.022*
Duration of alternative medicine	1.185 (1.111–1.263)	p<0.001*
Number of visits for alternative medicine	3.815 (2.217–6.564)	p<0.001*
Cost for alternative medicine	1.000 (1.000–1.000)	p<0.001*

CI, Confidence intervals.

*Significant difference between a low cost group (lower third) and a high cost group (upper third) (p < 0.05).

**Table 3:** Multivariate logistic regression analysis for the high-cost groups.

	Odds ratio (95%CI)	p-value
*Demographics*		
Age (group)		
17 ≤	0.250 (0.216–0.290)	p<0.001*
18–39	0.787 (0.748–0.828)	p<0.001*
40–59	1.084 (1.033–1.138)	p = 0.001*
Female	1.064 (1.022–1.108)	p = 0.003*
Occupation		
Salaried worker	1.007 (0.958–1.058)	p = 0.785
Homemaker	1.514 (1.419–1.616)	p<0.001*
Student	0.569 (0.500–0.648)	p<0.001*
A history of WAD in traffic accident	2.183 (2.033–2.344)	p<0.001*
Resident area		
Urban	1.466 (1.400–1.536)	p<0.001*
Suburban	1.142 (1.093–1.193)	p<0.001*
*Traffic accident-related variables*		
Collision types		
Rear-end collision	0.733 (0.689–0.779)	p<0.001*
Contact with one moving in the opposite direction	0.932 (0.864–1.006)	p = 0.069
Contact with a vehicle moving in the same direction	0.962 (0.875–1.056)	p = 0.414
Engine size of the other vehicle	1.000 (1.000–1.000)	p = 0.192
A responsibility of the patient	1.295 (1.210–1.385)	p<0.001*
*Treatment related-variables*		
Time to first visit for conventional medicine	0.998 (0.996–1.000)	p = 0.111
Multiple doctor visits	2673.269 (1002.247–7130.343)	p<0.001*
Alternative medicine	694.289 (436.070–1105.414)	p<0.001*

CI, Confidence intervals.

*Significant difference between a low cost group (lower third) and a high cost group (upper third) (p < 0.05).

The total weight of the other vehicle involved was recorded in 68630 patients (S2 Table). There was no significant difference between the low- and high-cost groups in this respect (p = 0.331). The correlation coefficient between the total weight of the other vehicle and its engine size was 0.891 (p<0.001, S2 Fig). A residual disability level of 14 was associated with occupation, a history of WAD claims, traffic accident- and treatment-related variables, and clinical and economic outcomes, compared to matched control subjects without residual disabilities (S3 Table).

Table 4 shows that the cost for consecutive medicine, for consecutive and alternative medicine and total healthcare costs were significantly associated with all clinical outcomes (CC>0.700).

**Table 4:** Correlation of economic outcome and clinical outcome.

	Cost for consecutive medicine (yen)		Cost for consecutive and alternative medicine (yen)		Total healthcare cost (yen)	
	Correlation coefficient	p-value	Correlation coefficient	p-value	Correlation coefficient	p-value
Number of visits for conventional medicine (days)	rs = 0.861	p<0.001*	rs = 0.795	p<0.001*	rs = 0.846	p<0.001*
Number of visits for conventional and alternative medicine (days)	rs = 0.782	p<0.001*	rs = 0.881	p<0.001*	rs = 0.942	p<0.001*
Duration of conventional medicine (days)	rs = 0.823	p<0.001*	rs = 0.766	p<0.001*	rs = 0.801	p<0.001*
Duration of conventional and alternative medicine (days)	rs = 0.732	p<0.001*	rs = 0.806	p<0.001*	rs = 0.842	p<0.001*
Time to compensation closure (days)	rs = 0.725	p<0.001*	rs = 0.798	p<0.001*	rs = 0.831	p<0.001*

*Significant correlation using Spearman’s rank correlation coefficient (p < 0.05)

Table 5 shows the differences between groups based on multiple doctor visits and visits for alternative medicine. Patients with multiple doctor visits and visits for alternative medicine showed a significantly high total healthcare cost per person (median, 292,346 yen; p<0.001) and a long time to compensation closure (median, 72 days; p<0.001).

**Table 5:** Difference between groups based on multiple doctor visits and visit for alternative medicine.

	WITHOUT multiple doctor visits, WITHOUT alternative medicine	WITH multiple doctor visits, WITHOUT alternative medicine	WITHOUT multiple doctor visits, WITH alternative medicine	WITH multiple doctor visits, WITH alternative medicine	
	(n = 89,024)	(n = 8,152)	(n = 6,986)	(n = 749)	p-value
*Demographics*					
Age (years)	43 [28–57]	41 [27–54]	36 [24–48]	36 [25–47]	p<0.001*
Age (group), n (%)					
17 ≤	9,804 (11%)	711 (9%)	791 (11%)	82 (11%)	p<0.001*
18–39	29,145 (33%)	3,209 (39%)	3,201 (46%)	343 (46%)	
40–59	30,857 (35%)	2,762 (34%)	2,277 (33%)	261 (35%)	
≥ 60	19,218 (21%)	1,470 (18%)	717 (10%)	63 (8%)	
Gender, n (%)					
Female	40,686 (46%)	3,775 (46%)	3,160 (45%)	359 (48%)	p = 0.351
Male	48,338 (54%)	4,377 (54%)	3,826 (55%)	390 (52%)	
Occupation, n (%)					
Salaried worker	48,486 (54%)	4,611 (57%)	4,098 (59%)	414 (55%)	p<0.001*
Homemaker	13,636 (15%)	1,128 (14%)	814 (12%)	70 (9%)	
Student	11,514 (13%)	935 (11%)	973 (14%)	107 (14%)	
Others	15,388 (17%)	1,478 (18%)	1,101 (16%)	158 (21%)	
A history of WAD in traffic accident, n (%)					
With	5,241 (6%)	532 (7%)	649 (9%)	77 (10%)	p<0.001*
Without	83,783 (94%)	7,620 (93%)	6,337 (91%)	672 (90%)	
Resident area, n (%)					
Urban	29,354 (33%)	2,837 (35%)	2,412 (35%)	274 (37%)	p<0.001*
Suburban	36,370 (41%)	3,249 (40%)	2,687 (38%)	292 (39%)	
Rural	23,300 (26%)	2,066 (25%)	1,887 (27%)	183 (24%)	
*Traffic accident related-variables*					
Collision types, n (%)					
Rear-end collision	64,026 (72%)	5,988 (73%)	4,494 (64%)	509 (68%)	p<0.001*
Contact with one moving in the opposite direction	11,174 (13%)	1,055 (13%)	1,033 (15%)	102 (14%)	
Contact with a vehicle moving in the same direction	4,704 (5%)	398 (5%)	459 (7%)	56 (7%)	
Others	9,120 (10%)	711 (9%)	1,000 (14%)	82 (11%)	
Engine size of the other vehicle (cc)	1,348 [658–1,997]	1,394[658–1,998]	1,368 [658–1,997]	1,496 [658–1,998]	p = 0.007*
A responsibility of the patient, n (%)					
With	10,850 (12%)	1,009 (12%)	1,109 (16%)	108 (14%)	p<0.001*
Without	78,174 (88%)	7,143 (88%)	5,877 (84%)	641 (86%)	
*Treatment related variables*					
Time to first visit for conventional medicine (days)	1 [0–2]	0 [0–1]	1 [0–3]	0 [0–1]	p<0.001*
*Clinical outcome*					
Number of visits for conventional medicine (days)	2 [1–5]	7 [4–13]	2 [1–3]	4 [3–10]	p<0.001*
Number of visits for conventional and alternative medicine (days)	2 [1–5]	7 [4–13]	12 [7–19]	16 [9–27]	p<0.001*
Duration of conventional medicine (days)	7 [1–30]	43 [21-80]	3 [1–23]	41 [16-148]	p<0.001*
Duration of conventional and alternative medicine (days)	9 [2–32]	40 [20-75]	46 [28-71]	66 [38-133]	p<0.001*
Time to compensation closure (days)	15 [9–38]	46 [26-80]	51 [34-77]	72 [43-136]	p<0.001*
Chronicity, n (%)					
With	4,140 (5%)	1,499 (18%)	977 (14%)	259 (35%)	p<0.001*
Without	84,884 (95%)	6,653 (82%)	6,009 (86%)	490 (65%)	
*Economic outcome*					
Cost for conventional medicine (yen)	33,379 [23,148–62,604]	95,279 [66,939–138,479]	31,121 [23,752–46,763]	78,578 [55,538–132,096]	p<0.001*
Cost for conventional and alternative medicine (yen)	33,379 [23,148–62,604]	95,279 [66,939–138,479]	90,639 [64,229–122,335]	136,625 [100,534–202,095]	p<0.001*
Total healthcare cost (yen)	53,587 [34,445–123,350]	175,692 [112,194–277,885]	215,549 [137,102–303,579]	292,346 [201,521–388,390]	p<0.001*
(Only patients with alternative medicine (n = 7,735))					
Time to first visit for alternative medicine (days)	0 [0–0]	0 [0–0]	6 [3–13]	9 [4–20]	
Duration of alternative medicine (days)	0 [0–0]	0 [0–0]	34 [17–57]	36 [16-70]	
Number of visits for alternative medicine (days)	0 [0–0]	0 [0–0]	10 [5–16]	10 [4–19]	
Cost for alternative medicine (yen)	0 [0–0]	0 [0–0]	51,485 [30,840–79,103]	51,120 [28,555–87,55]	

CI, Confidence intervals.

Data from continuous variables are shown in medians and interquartile ranges [IQR]. Data from categorical variables are shown in number and (%) of patients.

*Significant difference among groups using the Kruskal-Wallis test or chi-square test (p < 0.05).

## Discussion

The present study showed, for the first time, that multiple doctor visits and visits for alternative medicine were strong predictive factors of a high total healthcare cost per person in patients with acute WAD in no-fault government insurance in Japan. Patients with multiple doctor visits and visits for alternative medicine showed a high total healthcare cost per person and a long time until compensation for closure.

Half of the population with the lowest health spending represented <3% of the total healthcare costs [32]. Approximately 35% of direct costs are concentrated among the 4%, defined as persistent high-cost patients with musculoskeletal pain [18]. The high-cost patients are reportedly associated with chronic and mental illness, increasing age, and higher income [15]. Psychological well-being generally had U-shaped age profiles, with the lowest levels in the 50s [33]. In addition, a study reported that females have higher medical care service utilization and higher associated charges than males [34]. Frequently compensated services in acute WAD are emergency services, radiology services, and medical specialists, and physiotherapy, general practitioners, and radiology services in chronic WAD [22]. Female patients with WAD receive more payments for physiotherapists, chiropractors, and psychologists, whereas males receive more medical services payments for medical specialists, emergency services, and radiology services [22]. Consistent with previous population studies, male sex was identified as an independent predictive factor for low cost in the present study [21, 22]. Further, the total healthcare cost per person was significantly associated with clinical outcomes. There was no obvious association of outcomes with the traffic accident-related variables in acute WAD patients in the economic outcomes (current study) or clinical outcomes [9]. Predictive indicators relevant to poor clinical outcome of acute WAD are pain levels, disability, and physical and psychological factors in guidelines [35], similar to previous studies in Japan [9, 36, 37].

Overtreatment in the initial phase after acute WAD associated with slow recovery [7, 8, 10, 24]. Reliance on clinical care may have a negative effect on recovery by promoting the use of passive coping strategies [38, 39]. Japanese patients can visit medical institutions freely under the national health system, leading to a high prevalence of doctor-shopping [40]. Doctor-shopping, visiting multiple doctors for the same health problem, involves overlapping prescriptions and increased medical expenses. In the present study, patients with multiple doctor visits and visits for alternative medicine showed a significantly high total healthcare cost and a long time until compensation closure. Doctor-shopping patients in Japan are characterized by illness chronicity, their inability to understand doctors’ explanations, disbelief of doctor’s diagnosis and treatment, and high psychological disturbances [41]. Collision type and engine size of the other involved vehicle exhibited no consistent association with the total healthcare cost in the present study. Accordingly, doctor-shopping may be influenced by patients’ preferences, not injury severity.

The recommended first-line treatment for acute WAD is range-of-motion, low-load isometric, postural endurance, and strengthening exercises; staying active; and returning to usual activities [34]. Manual therapy, thoracic manipulation, acupuncture, kinesio taping, trigger point needling, and surgical treatment are not routinely recommended [34]. The proportion of individuals receiving recommended first-line care for various conditions was reported to be 54.9% [42]. The guideline of acute WAD recommends X-ray investigations and active treatments; however, some practices are not compliant [43]. The seniority of the first-treating physician did not influence the outcome of acute WAD [44]. A total of 9.6% of general practitioners are deemed to have low knowledge of WAD [45] and 23.4% of physical therapists report non-familiarity with evidence-based or clinical practice guidelines for treating patients with WAD [46]. Over half of the pharmaceutical claimants are prescribed non-steroidal anti-inflammatory drugs and weak opioid medicines, and over one-quarter are prescribed benzodiazepines in acute and chronic WAD [47]. Similar to our findings in Japan, the most frequently chosen type of treatment for chronic musculoskeletal pain is massage (31%), followed by medication (22%), physical therapy (16%), and acupuncture (9%) [26]. Twenty-nine percent of the higher-quality studies show a health improvement with cost savings for complementary and integrative medicine therapy versus usual care [48]. Exercise and manual therapy are core treatments in patients with neck pain provided by both conventional and alternative medicine; however, there are differences between chiropractors and physical therapists [49]. The major reasons for choosing alternative medicine are benefit expectations, dissatisfaction with conventional medicine, and its perceived safety [50]. The degree of satisfaction with the initial alternative medicine treatment for chronic musculoskeletal pain is higher than conventional medicine [51]. Asian populations more frequently report using alternative medicine than Western populations because they are influenced by members of their social network, the low cost and easy access to alternative medicine, and tradition [50]. On the other hand, Western populations mainly report satisfaction with conventional medicine or have never considered alternative medicine [50]. Treatment, cost benefit, cost effectiveness, and cost utility analysis have not been investigated in WAD.

The prevalence of WAD continues to increase, particularly in developed countries [52]. Meanwhile, the health service cost of WAD has decreased in Australia [21, 22], Sweden [53], and Canada [54]. The number of healthcare visits for pain significantly decreased in non-cancer pain in the United States [55]. The frequency and recovery of WAD may be influenced by cultural conditions and symptom expectations [6, 56–61], legal and insurance factors [54, 62–64], and awareness through media [21, 53]. Similarly, the present study showed that areas with a high unemployment rate, a high number of traffic accidents, and persons killed or injured were associated with a high total healthcare cost per person. Mass media campaigns for pain in seven countries appear to be effective for improving beliefs of the general public and healthcare providers, making beliefs more in line with current evidence and self-management principles [65].

The present study had several limitations. First, it included only a category in the General Insurance Rating Organization of Japan. The other, a unique insurance service system initiated by the Japan Agricultural Cooperatives group, mostly provides insurance for farmers in rural areas. Second, the medical expenses of car accident casualties in Japan may include not only those covered by public car insurance but also those covered by public and private medical insurance, and some patients may finalize their claims despite incomplete recovery [66]. Third, we did not investigate the symptoms, other diagnosis, traffic situation details, content of treatment, cost of indirect healthcare, or specific cost analysis. We only investigated the total cost of WAD. Of the annual economic cost of pain, half is for indirect healthcare [12]. The average societal costs of WAD during the first 4 months were reported as £99.55 for patients with no disability, increasing to £668.53 for those with complete disability [67, 68]. Diseases of the musculoskeletal system and connective tissue constitute a large portion of the cost burden due to presenteeism in Japan [69]. Fourth, pre-existing problems were not included in the database; the insurance covers only injury from the automobile crash based on the doctor’s diagnosis, and some WAD symptoms may mix with pre-existing pain problems. Fifth, the database excluded patients whose claim had not been closed. These patients may have a high healthcare utilization. Finally, there was no data on how long the patients needed to search for a second doctor or alternative medicine. The loss of treatments between treatments may have an impact on outcomes.

## Conclusions

Our results show that a high total healthcare cost is strongly associated with multiple doctor visits and visits for alternative medicine in patients with WAD in no-fault government insurance in Japan. Based on our findings, adequate interventions should be targeted and tailored to high-need patients that are most likely to benefit.

## Supporting information

S1 Fig Total healthcare cost per person in each resident prefecture. The provinces of Kanto, Tohoku, and Kyushu had a prefecture with a high total healthcare cost. This study was approved by the Ethics Committee of the Osaka University Graduate School of Medicine (No. 17136).
(TIF)

S2 Fig Correlation between total weight of the other vehicle and its engine size. This study was approved by the Ethics Committee of the Osaka University Graduate School of Medicine (No. 17136).
(TIF)

S1 Table Factors associated with total healthcare cost per person among 47 prefecture. (DOCX)

S2 Table Difference of total weight of the other vehicle between groups based on total healthcare cost. (DOCX)

S3 Table Difference between the subjects with and without the residual disability. (DOCX)
