# Supplementary figures and images for: Predictors of high-cost patients with acute whiplash-associated disorder in Japan
Source: PLoS One. 2023 Jun 28;18(6):e0287676. doi: 10.1371/journal.pone.0287676 (PMC10306225; doi:10.1371/journal.pone.0287676)

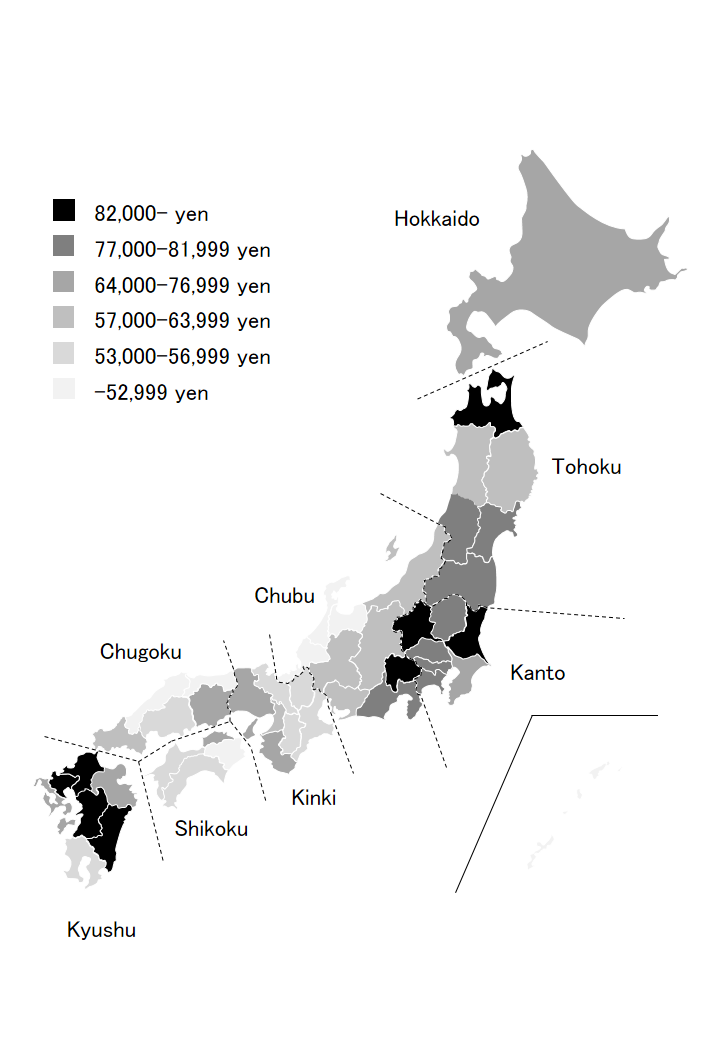

Supplement: S1 Fig — The provinces of Kanto, Tohoku, and Kyushu had a prefecture with a high total healthcare cost. This study was approved by the Ethics Committee of the Osaka University Graduate School of Medicine (No. 17136). (TIF) [file pone.0287676.s001.tif]

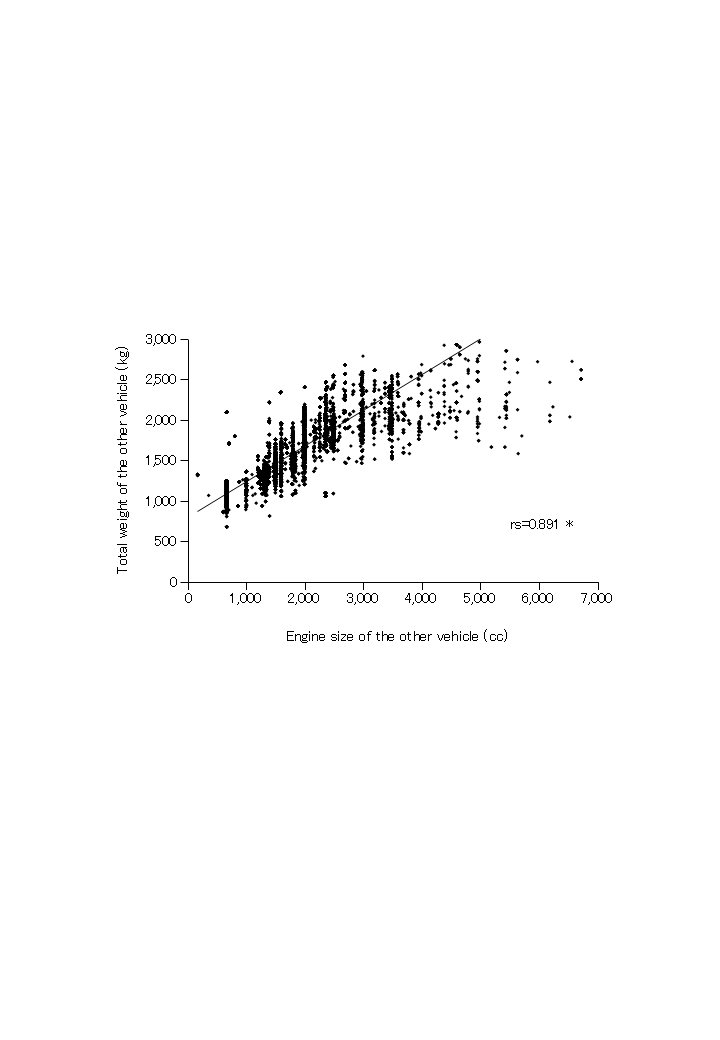

Supplement: S2 Fig — This study was approved by the Ethics Committee of the Osaka University Graduate School of Medicine (No. 17136). (TIF) [file pone.0287676.s002.tif]
