# Supplementary material for: Predictors of high-cost patients with acute whiplash-associated disorder in Japan
Source: PLoS One. 2023 Jun 28;18(6):e0287676. doi: 10.1371/journal.pone.0287676 (PMC10306225; doi:10.1371/journal.pone.0287676)
Supplement: S1 Table — (DOCX) [file pone.0287676.s003.docx]

**Supplementary Table 1.** Factors associated with total healthcare cost per person among 47 prefecture

|  | Correlation coefficient | p-value |
| --- | --- | --- |
| *Prefectures' characteristics* |  |  |
| Population per 1 km^2^ of total land area (persons) | rs=0.183 | p=0.219 |
| Population per 1 km^2^ of inhabitable area (persons) | rs=0.054 | p=0.717 |
| Yearly average of air temperature (degree Celsius) | rs=0.049 | p=0.743 |
| Yearly sunshine hours (hours) | rs=0.247 | p=0.094 |
| Ratio of people having completed up to colleges and universities (%) | rs=-0.063 | p=0.676 |
| Recognitions of criminal offenses (number of cases per 1,000 persons) | rs=0.167 | p=0.262 |
| *Economic status* |  |  |
| Regional Difference Index of Consumer Prices (All items) | rs=-0.161 | p=0.279 |
| Prefectural income per person (thousand yen) | rs=0.023 | p=0.878 |
| Amount of savings per household (Two-or-more-person households) (thousand yen) | rs=-0.251 | p=0.089 |
| Monthly income per household (workers' households of two-or-more-person households) (thousand yen) | rs=-0.097 | p=0.518 |
| Ratio of persons employed in the primary industry (%) | rs=0.084 | p=0.577 |
| Ratio of persons employed in the secondary industry (%) | rs=-0.056 | p=0.711 |
| Ratio of persons employed in the tertiary industry (%) | rs=-0.071 | p=0.635 |
| Unemployment rate (%) | rs=0.300 | p=0.040* |
| *Medical status* |  |  |
| National medical expenses per person (thousand yen) | rs=-0.172 | p=0.248 |
| General hospitals (hospitals per 100,000 persons) | rs=-0.159 | p=0.287 |
| General clinics (clinics per 100,000 persons) | rs=-0.193 | p=0.193 |
| General hospitals (hospitals per 100km^2^ of inhabitable area) | rs=-0.031 | p=0.835 |
| General clinics (clinics per 100km^2^ of inhabitable area) | rs=-0.024 | p=0.875 |
| *Traffic status* |  |  |
| Traffic accidents (number of cases per 100,000 persons) | rs=0.473 | p=0.001* |
| Persons killed or injured by traffic accidents (persons per 100,000 persons) | rs=0.481 | p=0.001* |

*Significant correlation using Spearman’s rank correlation coefficient (p < 0.05)

This study was approved by the Ethics Committee of the Osaka University Graduate School of Medicine (No. 17136).
