# Supplementary material for: Predictors of high-cost patients with acute whiplash-associated disorder in Japan
Source: PLoS One. 2023 Jun 28;18(6):e0287676. doi: 10.1371/journal.pone.0287676 (PMC10306225; doi:10.1371/journal.pone.0287676)
Supplement: S2 Table — (DOCX) [file pone.0287676.s004.docx]

**Supplement Table 2.** Difference of total weight of the other vehicle between groups based on total healthcare cost

|  | Overall | Low cost | Medium cost | High cost |  |
| --- | --- | --- | --- | --- | --- |
|  | (n=68,630) | (n=22,927) | (n=22,765) | (n=22,938) | p-value |
| Total weight of the other vehicle (kg) | 1,285 [1,090-1,655] | 1,275 [1,090-1,655] | 1,285 [1,090-1,655] | 1,285 [1,090-1,655] | p=0.331 |

CI, Confidence intervals.

Data from continuous variables are shown in medians and interquartile ranges [IQR]. There was no significant difference between a low cost group (lower third) and a high cost group (upper third).

The correlation coefficient between total weight of the other vehicle and its engine size was 0.891 (p<0.001).

This study was approved by the Ethics Committee of the Osaka University Graduate School of Medicine (No. 17136).
