# Supplementary material for: Predictors of high-cost patients with acute whiplash-associated disorder in Japan
Source: PLoS One. 2023 Jun 28;18(6):e0287676. doi: 10.1371/journal.pone.0287676 (PMC10306225; doi:10.1371/journal.pone.0287676)
Supplement: S3 Table — (DOCX) [file pone.0287676.s005.docx]

**Supplement Table 3.** Difference between the subjects with and without the residual disability.

|  | Without the residual disability (matched control subjects) | With the residual disabilities level 14 | Univariate |  |
| --- | --- | --- | --- | --- |
|  | (n=248) | (n=248) | Odds ratio (95%CI) | p-value |
| *Demographics* | | |  |  |
| Age (group), n (%) |  |  |  |  |
| 17 ≤ | 3 (1%) | 0 (0%) | 0.000 (0.000-0.000) | p=0.999 |
| 18–39 | 85 (34%) | 69 (28%) | 0.739 (0.505-1.083) | p=0.121 |
| 40–59 | 132 (53%) | 144 (58%) | 1.217 (0.853-1.735) | p=0.278 |
| ≥ 60 | 28 (11%) | 35 (14%) | 1.291 (0.759-2.197) | p=0.346 |
| Gender, n (%) |  |  |  |  |
| Female | 109 (44%) | 88 (35%) | 0.701 (0.489-1.007) | p=0.054 |
| Male | 139 (56%) | 160 (65%) |  |  |
| Occupation, n (%) |  |  |  |  |
| Salaried worker | 143 (58%) | 114 (46%) | 0.625 (0.438-0.891) | p=0.009* |
| Homemaker | 34 (14%) | 18 (7%) | 0.493 (0.270-0.898) | p=0.021* |
| Student | 4 (2%) | 1 (0%) | 0.247 (0.027-2.225) | p=0.212 |
| Others | 67 (27%) | 115 (46%) | 2.336 (1.605-3.400) | p<0.001* |
| A history of WAD in traffic accident, n (%) | |  |  |  |
| With | 32 (13%) | 56 (23%) | 1.969 (1.223-3.168) | p=0.005* |
| Without | 216 (87%) | 192 (77%) |  |  |
| Resident area, n (%) | |  |  |  |
| Urban | 96 (39%) | 109 (44%) | 0.805 (0.563-1.152) | p=0.236 |
| Suburban | 93 (38%) | 100 (40%) | 0.888 (0.619-1.274) | p=0.519 |
| Rural | 59 (24%) | 41 (17%) | 1.576 (1.010-2.459) | p=0.045* |
|  |  |  |  |  |
| *Traffic accident related-variables* | |  |  |  |
| Collision types, n (%) | |  |  |  |
| Rear-end collision | 131 (53%) | 139 (56%) | 1.139 (0.800-1.622) | p=0.471 |
| Contact with one moving in the opposite direction | 60 (24%) | 52 (21%) | 0.831 (0.545-1.267) | p=0.391 |
| Contact with a vehicle moving in the same direction | 12 (5%) | 27 (11%) | 2.403 (1.188-4.859) | p=0.015* |
| Others | 45 (18%) | 30 (12%) | 0.621 (0.377-1.023) | p=0.062 |
| Engine size of the other vehicle (cc) | 1496 [658-1,998] | 1,498 [658-1,998] | 1.000 (1.000-1.000) | p=0.040* |
| A responsibility of the patient, n (%) | |  |  |  |
| With | 68 (27%) | 62 (25%) | 0.882 (0.591-1.317) | p=0.540 |
| Without | 180 (73%) | 186 (75%) |  |  |
|  |  |  |  |  |
| *Treatment related-variables* |  |  |  |  |
| Time to first visit for conventional medicine (days) | 1 [0-1] | 0 [0-1] | 0.882 (0.784-0.993) | p=0.039* |
| Multiple doctor visits, n (%) |  |  |  |  |
| With | 151 (61%) | 185 (75%) | 1.886 (1.286-2.767) | p=0.001* |
| Without | 97 (39%) | 63 (25%) |  |  |
| Alternative medicine, n (%) |  |  |  |  |
| With | 66 (27%) | 55 (22%) | 0.786 (0.521-1.185) | p=0.251 |
| Without | 182 (73%) | 193 (78%) |  |  |
|  |  |  |  |  |
| *Clinical outcome* |  |  |  |  |
| Number of visits for conventional medicine (days) | 49 [20-69] | 70 [50-102] | 1.019 (1.013-1.024) | p<0.001* |
| Number of visits for conventional and alternative medicine (days) | 62 [43-85] | 88 [61-112] | 1.017 (1.011-1.022) | p<0.001* |
| Duration of conventional medicine (days) | 183 [158-219] | 197 [180-227] | 1.003 (1.000-1.006) | p=0.028* |
| Duration of conventional and alternative medicine (days) | 184 [162-219] | 200 [181-233] | 1.000 (0.999-1.000) | p=0.599 |
| Time to compensation closure (days) | 187 [164-220] | 199 [182-225] | 1.003 (1.000-1.006) | p=0.053 |
| Chronicity, n (%) |  |  |  |  |
| With | 235 (95%) | 243 (98%) | 0.372 (0.131-1.060) | p=0.064 |
| Without | 13 (5%) | 5 (2%) |  |  |
|  |  |  |  |  |
| *Economic outcome* |  |  |  |  |
| Cost for conventional medicine (yen) | 406,679 [250,887-546,555] | 443,938 [312,310-561,293] | 1.000 (1.000-1.000) | p=0.963 |
| Cost for conventional and alternative medicine (yen) | 488,060 [369,920-622,239] | 487,765 [369,844-621,797] | 1.000 (1.000-1.000) | p=0.182 |
| Total healthcare cost (yen) | 848,418 [575,394-1,041,201] | 750,000 [599,468-834,887] | 1.000 (1.000-1.000) | p=0.005* |

CI, Confidence intervals.

Data from continuous variables are shown in medians and interquartile ranges [IQR]. Data from categorical variables are shown in number and (%) of patients. *Significant difference between the subjects with and without the residual disability (p < 0.05).

This study was approved by the Ethics Committee of the Osaka University Graduate School of Medicine (No. 17136).
